# Exosomes: potential diagnostic markers and drug carriers for adenomyosis

**DOI:** 10.3389/fphar.2023.1216149

**Published:** 2023-08-23

**Authors:** Wen-Xiu Cheng, Shao-Bin Wei, Yang Zhou, Yu Shao, Mao-Ya Li

**Affiliations:** ^1^ Department of Gynecology, Hospital of Chengdu University of Traditional Chinese Medicine, Chengdu, Sichuan, China; ^2^ Trauma Center, Rizhao Hospital of Traditional Chinese Medicine, Rizhao, Shandong, China

**Keywords:** adenomyosis, pathogenesis, exosomes, cell proliferation, fibrosis formation

## Abstract

Adenomyosis is a common benign gynecological disorder and an important factor leading to infertility in fertile women. Adenomyosis can cause deep lesions and is persistent and refractory in nature due to its tumor-like biological characteristics, such as the ability to implant, adhere, and invade. The pathogenesis of adenomyosis is currently unclear. Therefore, new therapeutic approaches are urgently required. Exosomes are nanoscale vesicles secreted by cells that carry proteins, genetic materials and other biologically active components. Exosomes play an important role in maintaining tissue homeostasis and regulating immune responses and metabolism. A growing body of work has shown that exosomes and their contents are key to the development and progression of adenomyosis. This review discusses the current research progress, future prospects and challenges in this emerging therapeutic tool by providing an overview of the changes in the adenomyosis uterine microenvironment and the biogenesis and functions of exosomes, with particular emphasis on the role of exosomes and their contents in the regulation of cell migration, proliferation, fibrosis formation, neovascularization, and inflammatory responses in adenomyosis.

## 1 Introduction

Adenomyosis (AM) is a benign gynecological disorder characterized by diffuse or limited hypertrophic hyperplasia due to invasion of the myometrium by the endometrial glands and mesenchyme, causing infertility in fertile women (Gordts et al., 2018). The typical clinical symptoms of AM are pelvic pain, abnormal uterine bleeding, and infertility, which seriously affect the physical and mental health of affected women (Abbott, 2017). In recent years, the prevalence of AM has been rising annually, especially in younger populations. Studies have shown that some women display typical symptoms and ultrasound features of AM as early as during their pre-reproductive period (Pinzauti et al., 2015; Upson and Missmer, 2020). Although the pathogenesis of AM remains unclear, it is thought to be associated with endometrial damage and involution, hormonal factors, myometrial stem cell metaplasia, and immunogenetic factors (Zhai et al., 2020). AM and Endometriosis (EM) were considered different manifestations of the same disease in the past. Since Franklin first proposed the concept of “adenomyosis” in 1925, people have gradually realized the difference between the two. However, because the pathological changes of both involve the ectopic endometrium, the symptoms of the two are similar, and the two often appear together in clinical practice, so the treatment methods also have similarities. However, compared with EM, AM-related research is less. A search of PubMed with the subject term “adenomyosis” identified only 1,603 articles as of December 31, while there were 19,019 EM articles in the same period. It can be seen that there is still a lot of research space for AM, which is worthy of further exploration by researchers.

For AM, western medical treatment is predominantly based on hormonal drugs, such as oral contraceptives, gonadotropin-releasing hormone agonists, levonorgestrel-releasing intrauterine system, mifepristone, and androgen derivatives. Although medical treatments are efficacious, they are associated with significant adverse effects and recurrence after discontinuation (Vannuccini et al., 2018). Total hysterectomy is the radical treatment for AM, but conservative surgery with uterus preservation is often performed for patients who want to preserve the uterus to maintain fertility. The latter surgical approach involves focal resection of AM lesions only, and is applicable to AM with limited lesions. However, focal resection is not suitable for diffuse AM with extensive lesions due to its inability to completely remove the lesions, and is associated with a high risk of recurrence (Grimbizis et al., 2014; Tsui et al., 2014). It can be seen that the current treatment of AM has its limitations. Seeking a new and effective treatment method is an urgent problem to be solved in the field of AM research.

Exosomes (EXOs) are small extracellular vesicles with a diameter of 30–150 nm derived from intracellular lysosomal particles that are invaginated and released into the extracellular matrix (ECM) after fusion with the cell membrane (Théry et al., 2006). EXOs contain a wide range of proteins, lipids and genetic materials, and their inherent ability to carry multiple active substances through cells and high affinity for target cells have gained increasing attention in recent years (Zhang et al., 2019b). Under physiological and pathological conditions, almost all cell types release EXOs for intercellular communication (Yang et al., 2020). EXOs have been reported to regulate various biological processes such as growth and development, tissue homeostasis, aging and metabolism under physiological conditions, and participate in the development and progression of inflammatory diseases, autoimmune diseases, and tumor-like diseases under pathological conditions (Milane et al., 2015; Zhang et al., 2019a; Pegtel and Gould, 2019; Mirzaei et al., 2021). It is reported that female reproductive tissues such as ovaries, fallopian tubes, endometrium, decidua, and placenta can produce EXOs (Foster et al., 2016; Simon et al., 2018). Due to these characteristics of EXOs, many researchers have found that EXOs play an important regulatory or therapeutic role in a variety of gynecological diseases, including cervical cancer (Fang et al., 2022), endometrial cancer (Fan J. T. et al., 2021), intrauterine adhesions (Ebrahim et al., 2018), EM (Zhou et al., 2020) and so on. More and more researchers have begun to explore the correlation between EXOs and AM. In this review, we provide an overview of studies focused on EXOs and the contents they carry, summarized the association and mechanism of action of EXOs and AM, and explored the potential research value and future prospects of EXOs in the diagnosis and treatment of AM.

## 2 Retrieval method

All relevant articles were retrieved from PubMed using the terms “exosomes”, “EVs”, “miRNAs”, “adenomyosis”, “endometriosis”, “mechanism”, “diagnosis”, and “therapy” from inception to December 2022, and were screened based on whether the studies have investigated the association between EXOs and AM.

## 3 EXOs

### 3.1 Biogenesis and composition of EXOs

**FIGURE 1:**
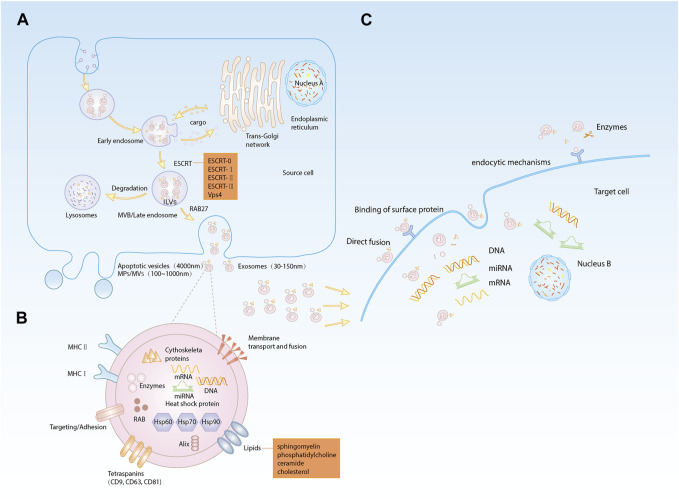
**(A)**. Biogenesis of EXOs: (a) plasma membrane invagination to form cup-like structures; (b) early endosomes formation; (c) early endosomes mature into late endosomes or MVBs; (d) degradation of MVBs releases ILVs, i.e., EXOs; **(B)**. Composition of EXOs: EXOs are lipid bilayer membrane structures carrying lipids, proteins, nucleic acids and other substances; **(C)**. Internalization of EXOs: (a) direct fusion; (b) binding to membrane protein receptors; (c) protein fragments bound to target cell membrane receptors after protease shearing; (d) mechanism of endocytosis.

Extracellular vesicles (EVs) are small membranous vesicles released from cells into the ECM and can be broadly classified as apoptotic vesicles (4,000 nm in diameter), microparticles/microvesicles (MPs/MVs) (100–1,000 nm in diameter) and EXOs (30–150 nm in diameter) (Porro et al., 2015). The first two types of vesicles can be released directly from the plasma membrane of the cell, while the biogenesis of EXOs involves double invagination of the plasma membrane and lysosomal degradation. This process can be divided into four stages: 1) Formation of a cup-like structure containing cell surface proteins and soluble proteins through invagination of the plasma membrane; 2) Formation of early endosomes (EEs) from the trans-Golgi network and endoplasmic reticulum; 3) Maturation of EEs into late endosomes (LEs) or multivesicular bodies (MVBs) through carrier selection mechanisms; and 4) Degradation of MVBs by fusion with lysosomes or autophagosomes or fusion of MVBs with the plasma membrane to release intraluminal vesicles (ILVs, which will turn into EXOs) (Raposo and Stoorvogel, 2013; Hessvik and Llorente, 2018; Kalluri and LeBleu, 2020). The endosomal sorting complex for transport (ESCRT) machinery is a key mediator of EXOs biogenesis. Components of ESCRT can bind to transmembrane cargoes in endosomes and sort them into EXOs (Katzmann et al., 2001; Henne et al., 2013). ESCRT consists of five complexes: ESCRT-0 is an ubiquitinated complex that aggregates cargoes and initiates the cargo sorting pathway; ESCRT-I, ESCRT-II, and ESCRT-III direct the budding of ILVs; and the Vps4 complex promotes ESCRT-III-mediated membrane fission (Henne et al., 2011; Juan and Fürthauer, 2018). In addition, other accessory proteins of ESCRT, such as Tsg101 and Alix, are also involved in formation and release of EXOs. These complexes recognize and sort ubiquitinated cargoes through precise partitioning. However, it was found that maximal inhibition of the ESCRT machinery in mammalian cells does not prevent the formation of EXOs (Stuffers et al., 2009). Thus, the biogenesis of EXOs may involve both ESCRT-dependent and ESCRT-independent mechanisms. ESCRT-independent EXOs biogenesis is closely related to the role of lipids and proteins. Trajkovic first reported ESCRT-independent biogenesis of proteolipid protein (PLP)-containing EXOs in oligodendrocytes. This mechanism of formation was dependent on ceramide, a lipid that facilitates the inward budding of ILVs by inducing lipid aggregation (Trajkovic et al., 2008). It has been shown that the RAB protein family can control the basic functions of vesicles by recruiting specific effector proteins (Galvez et al., 2012). RAB5 is now known to be involved in the regulation of EEs formation and fusion and is also a facilitator of EEs maturation into LEs (Simonsen et al., 1998). RAB31 can drive the formation of EXOs as ILVs in MVBs (Wei et al., 2021). The conversion from RAB5 to RAB7 allows MVBs to fuse with lysosomes and autophagosomes (Huotari and Helenius, 2011), and RAB27 (including RAB27a and RAB27b) regulates the release of ILVs from MVBs upon fusion to the plasma membrane (Ostrowski et al., 2010) (Figure 1A).

The biogenesis of EXOs is diverse, but regardless of the pathways of EXOs formation and release, EXOs are cellular vesicles with a lipid bilayer membrane enriched in lipids, proteins, nucleic acids, and other active substances from cells. EXOs membrane proteins include membrane transport- and fusion-related proteins, adhesion factors, antigen presentation-related proteins (e.g., MHC-II), tetraspanins (including CD9, CD63 and CD81), and heat shock proteins (including HSP60, HSP70 and HSP90), which are involved in targeted cellular transport and adhesion as well as T-cell activation (Zhang et al., 2018a; Jeppesen et al., 2019; Zhu et al., 2021). On the other hand, internal EXOs proteins include cytoskeletal proteins (including actin and microtubulin), ESCRT complexes, RAB family, enzymes and other cytoplasmic proteins (Zhang et al., 2018a; Jeppesen et al., 2019; Kalluri and LeBleu, 2020). The membrane of EXOs is mainly composed of lipids, including sphingomyelin, phosphatidylcholine, cholesterol and ceramide (Trajkovic et al., 2008; van Meer et al., 2008; Skotland et al., 2017; Skotland et al., 2019), which are mainly involved in signal transduction. EXOs carry specific nucleic acids such as DNA, mRNAs and noncoding RNAs, among which miRNAs are delivered to target cells as the main cargoes carried by EXOs to exert their corresponding regulatory role (Cheng et al., 2014) (Figure 1B).

### 3.2 Uptake and internalization of EXOs

There is increasing evidence demonstrating that EXOs are released into the intercellular space and then taken up by recipient cells. It has been found that the uptake and internalization of EXOs are accomplished through four pathways (Mulcahy et al., 2014). First, the EXOs membrane fuses directly with the target cell membrane (Parolini et al., 2009). Second, the membrane protein of EXOs can bind to the membrane protein receptor on the target cell, which in turn activates signaling pathways in the target cell (Rana and Zöller, 2011; Record et al., 2014). Third, EXOs membrane proteins can be cleaved by proteases in the ECM, and the cleaved protein fragments can bind to receptors on the target cells. Last, EXOs are taken up by the recipient cells through endocytic mechanisms, including clathrin-dependent endocytosis, caveolae-dependent endocytosis, lipid raft-mediated endocytosis, phagocytosis and macropinocytosis (Doherty and McMahon, 2009). Upon uptake by the recipient cell, EXOs release their cargoes to mediate intercellular communication processes (Figure 1C).

### 3.3 Function of EXOs

**FIGURE 2:**
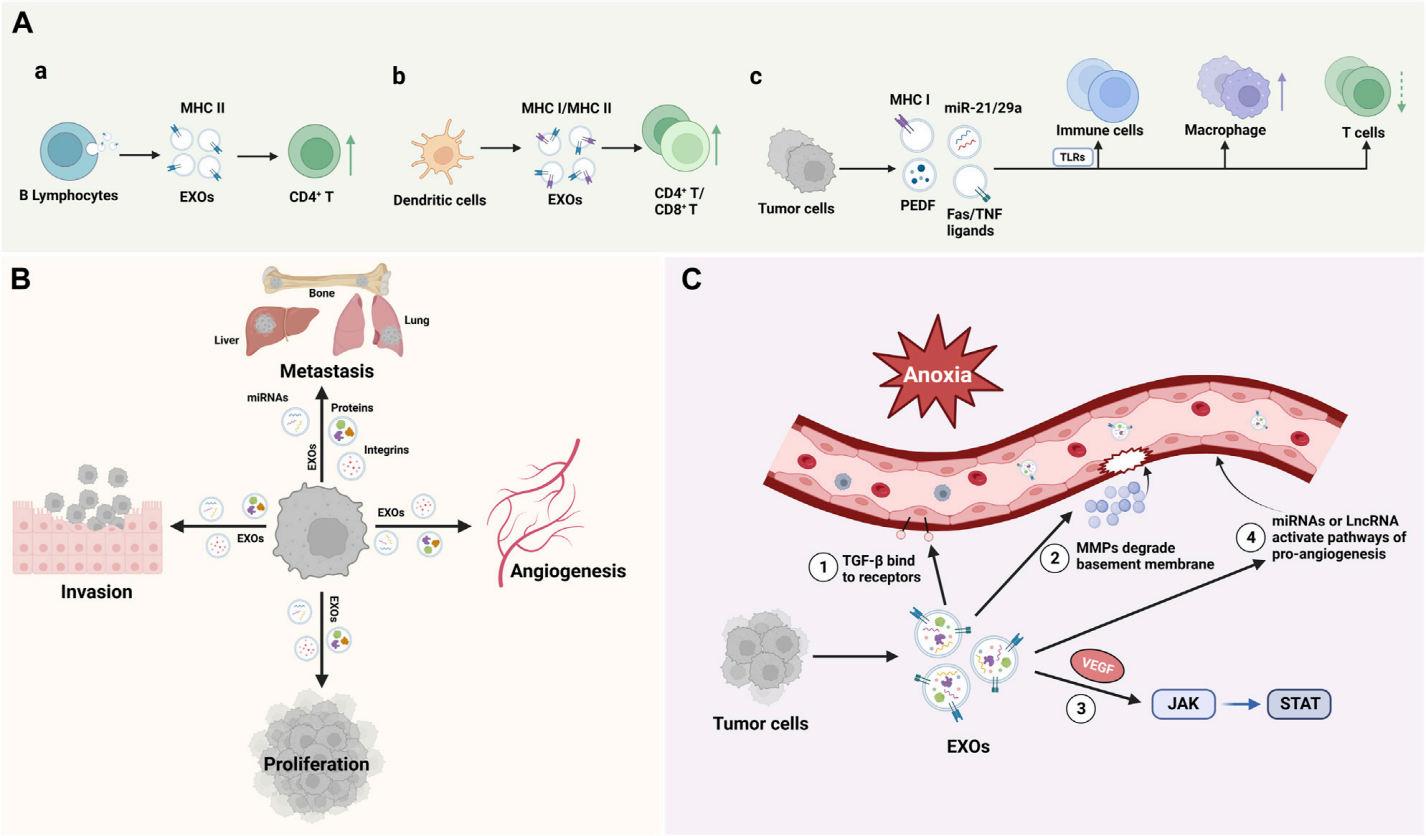
Function of EXOs: **(A)**. EXOs from distinct cellular sources, including B lymphocytes, dendritic cells and tumor cells, shed EXOs with cargos that can influence the innate and adaptive immune system; **(B)**. EXOs regulate cell Proliferation, invasion, and metastasis; **(C)**. EXOs promote angiogenesis. TGF-β, transforming growth factor-β; MMPs, matrix metalloproteinases; VEGF, vascular endothelial growth factor.

EXOs were first discovered in sheep reticulocytes in 1983 and were initially thought to be a form of cellular excretion (Pan and Johnstone, 1983). Though, as science and technology advance, EXOs have been found to perform various functions, the diversity of which depends mainly on the type of cells from which they originate. EXOs are naturally present in body fluids such as blood (Caby et al., 2005), saliva (Han et al., 2018), cerebrospinal fluid (Street et al., 2012), and urine (Pisitkun et al., 2004). Almost all types of cells release EXOs, and the contents of EXOs vary depending on the cell source. EXOs deliver their contents to recipient cells to mediate the corresponding biological response, and this EXOs-mediated response can participate in the development and progression of disease (Hessvik and Llorente, 2018). Recently, researchers have found that EXOs may be involved in biological processes such as immune response, cell migration, cell proliferation, and tumor invasion (Wang et al., 2019b), and play an important role in the diagnosis and treatment of neoplastic diseases, neurodegenerative pathologies, autoimmune diseases, and infectious diseases (Figure 2).

#### 3.3.1 Immunomodulation

In 1996, EXOs began to attract the attention of immunologists for their role in antigen presentation. Epstein‒Barr virus (EBV)-transformed B lymphocytes can secrete EXOs, which carry MHC class II molecules and can present processed antigen fragments to CD4^+^ T cells during the initiation phase of an immune response, suggesting that EXOs are involved in the host’s adaptive immunity (Raposo et al., 1996). Dendritic cells (DCs) were also found to secrete EXOs bearing MHC class I complexes, which can promote CD8^+^ T cell-dependent antitumor immune responses in mice (Zitvogel et al., 1998). Toll like receptors (TLRs) are important components of the innate immune system and are the determinants of the recognition of microbial pathogens and the initiation of the immune system (Wicherska-Pawlowska et al., 2021). Tumor cells can induce TLR-mediated NF-κB activation and protumoral inflammatory process by secreting a large number of EXOs containing miRNA-21 and miRNA-29a and binding to TLR8 and TLR7 in immune cells, leading to tumor growth and metastasis (Fabbri et al., 2012). Plebanek et al. found that EXOs released from nonmetastatic melanoma promote the differentiation and polarization of macrophages and participate in the killing and phagocytosis of tumor cells. These EXOs that carry immunomodulatory factors may be related to PEDF, a tumor suppressor with potent antiangiogenic and anticancer effects (Craword et al., 2013; Plebanek et al., 2017). In addition, Veronica found that colorectal cancer cells can induce apoptosis in T cells by releasing EXOs carrying Fas ligands and TNF-related apoptosis-inducing ligands (Huber et al., 2005). Therefore, EXOs have a bidirectional regulatory effect on the immune system, which can result in immune activation or immunosuppression depending on the role of the factors they carry (Figure 2A).

#### 3.3.2 Cell proliferation, invasion, and metastasis

Studies have shown that a key factor in promoting tumor metastasis is the formation of a microenvironment conducive to tumor metastasis at a specific site, namely, pre-metastatic niche (PMN). EXOs secreted by tumor cells are the key mediators of PMN formation (Liu and Cao, 2016). Popularly speaking, tumor cells are “seeds”, PMNs at specific sites are equivalent to “soil”, and EXOs are similar to “fertilizers”, which optimize the environment for tumor cell colonization, growth and metastasis. Feng et al. found that ovarian cancer exosomal proteins can enhance the progression of metastasis of ovarian tumors, and identified tumorigenic miRNAs in ovarian cancer-derived EXOs, including miRNA-99a-5p, miRNA-21 and miRNA-940, which can promote the formation of PMN (Feng et al., 2019). Experimental evidence (Yuan et al., 2021) showed that breast cancer cell-derived EXOs plays an important role in promoting bone metastasis of breast cancer, which is related to the transfer of miRNA-21 to osteoclasts to form PMN. A proteomic study of EXOs in pancreatic cancer showed that EXOs specifically express 362 proteins that are known to play a role in cell proliferation, cell migration, and tumor metastasis (Emmanouilidi et al., 2019). In addition, EXOs derived from highly metastatic cell lines were found to carry proteins that play more potent roles in adhesion, invasion, growth, and metastasis (Yu et al., 2017). By analyzing the EXO proteomics in various tumor models, it was found that tumor cell-derived EXOs could also direct organ-specific metastatic implantation (organotropism) of tumor cells by expressing specific integrins that mediate fusion with target cells, such as the EXO integrins α_6_β_4_ and α_6_β_1_, which are associated with lung metastasis, and α_v_β_5_, which is associated with liver metastasis (Hoshino et al., 2015) (Figure 2B).

#### 3.3.3 Pro-angiogenesis

EXOs derived from endothelial cells have pro-angiogenic and immunomodulatory effects (Wortzel et al., 2019). Angiogenesis and inflammation are key processes during tumorigenesis, in which abnormal angiogenesis has a central role in tumor development, characterized by excessive production of vascular endothelial growth factor (VEGF) (Jászai and Schmidt, 2019). Tumor cells can trigger epithelial-mesenchymal transition (EMT), angiogenesis and immune escape through the release of EXOs (Xie et al., 2019). Hypoxia is one of the characteristics of tumor development, and the detection of proangiogenic factors (ANFs) enriched in tumor cell-derived EXOs under hypoxia suggests that tumor cells can activate several signaling pathways to promote angiogenesis and to regulate the tumor microenvironment through the secretion of EXOs. These factors include the transforming growth factor-β(TGF-β), VEGF, matrix metalloproteinases (MMPs), certain miRNAs, and long noncoding RNAs (lncRNAs), and the associated pathways are the TGF-β/Smad pathway, JAK-STAT pathway, and Wnt4/β-catenin pathway (Aslan et al., 2019). Studies showed that dysregulation of miRNAs can affect some key pathways involved in tumor progression, and EXOs, as the carrier of miRNAs, can mediate miRNAs to promote angiogenesis (Tiwari et al., 2018). Wang et al. found that miRNA-BART10 and miRNA-18a were overexpressed in nasopharyngeal carcinoma (NPC) tissues and participated in the angiogenesis of NPC by activating VEGF, while EXOs loaded with antagomiRNA-BART10-5p and antagomiRNA-18a could inhibit the angiogenesis of NPC (Wang et al., 2020a). These studies suggested that the regulation of EXOs on diseases is two-way, which mainly depends on the factors they carry. Using this feature can block the development of diseases and play a therapeutic role (Figure 2C).

### 3.4 Clinical trials of EXOs

In view of the regulatory effect of EXOs on physiological and pathological changes, the isolation and application of EXOs are developing towards clinical trials. Through literature review, the performance of EXOs as a means of diagnosis and treatment has been tested in several clinical trials. In a phase-I trial, the researchers purified EXOs from DCs, loaded MHC class I peptides, and injected them intradermally and subcutaneously to 15 melanoma patients. EXOs therapy was tolerated up to 21 months. During this period, no obvious toxic reaction was observed, and a few patients had mild inflammatory reaction at the injection site. One of them exhibited a specific melanoma antigen T cell-response and a reduction in tumour size. The clinical trial highlighted the feasibility of large scale EXOs production and the safety of EXOs therapy (Escudier et al., 2005). A non-randomized phase I/II clinical trial showed that EXOs derived from DCs pulsed with SART1 presents a strong potential as a vaccine for esophageal cancer, which is well tolerated and can regulate the patient’s immune response (Narita et al., 2015). In a phase-II trial, the potential of EXOs as biomarkers has also been confirmed. The experimental results showed that insulin resistance is associated with Alzheimer’s disease (AD). EXOs rich in neurons carry insulin signal mediators, which can be used as biomarkers of cerebral insulin resistance to track changes in cognitive ability in AD treatment (Mustapic et al., 2019). At present, a clinical trial on the safety and tolerance of inhaled MSC-EXOs for healthy volunteers is underway (NCT04313647). Another clinical trial to evaluate the safety and efficacy of MSC-EXOs in promoting the healing of large and refractory macular holes (MHs) is also in progress (NCT03437759).

## 4 Pathogenesis of AM

The uterus is an organ with a thick luminal wall, which consists of the perimetrium (outer layer, visceral peritoneum), myometrium (middle layer, composed of smooth muscles), and endometrium (inner layer, mucosa). Since there is a lack of an “intermediate buffer”, namely, a submucosa, between the myometrium and endometrium, these two layers are in direct contact with each other. The histological features of AM are the presence of endometrial glands or mesenchyme in the myometrium surrounded by smooth muscle hyperplasia. This process is similar to the metastatic process of tumors, involving endometrial invasion and adhesion, basalis layer injury, abnormal smooth muscle function and ectopic endometrial proliferation-apoptosis imbalance (Schrager et al., 2022). The pathogenesis of AM remains largely unknown, but two hypotheses have been proposed, namely, the endometrial invagination hypothesis and the metaplasia hypothesis (García-Solares et al., 2018).

**FIGURE 3:**
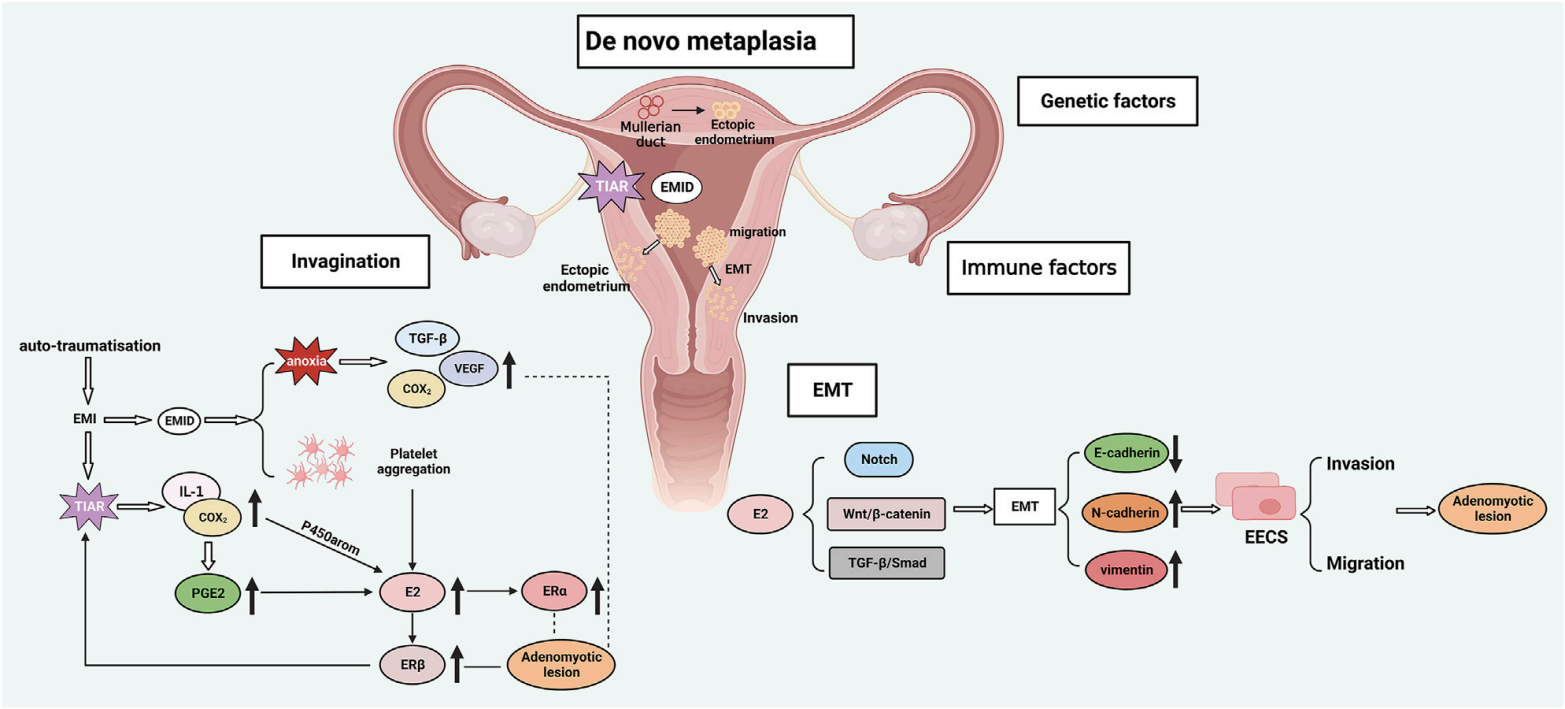
AM pathogenesis: A. Endometrial invagination: TIAR and EMID mechanisms; B. Metaplasia hypothesis; C. EMT; D. Genetic and immune factors. EMI, endometrial myometrial interface; EMID, endometrial-myometrial interface disruption; EMT, epithelial-mesenchymal transition; TIAR, tissue injury and repair; TGF-β, transforming growth factor-β; VEGF, vascular endothelial growth factor; IL-1, interleukin-1; COX2, cyclooxygenase-2; PGE2, prostaglandin E2; ERα, estradiol receptor α; ERβ, estradiol receptor β; P450 arom, aromatase cytochrome P450; EECs, endometrial epithelial cells.

Endometrial invagination mainly results from the activation of the tissue injury and repair (TIAR) mechanism (García-Solares et al., 2018). The TIAR mechanism was first proposed by Leyendecker et al., who found that chronic or excessive uterine peristalsis induces endometrial myometrial interface (EMI) microdamage. Activation of the TIAR mechanism triggers repair and inflammation, which in turn stimulates local IL-1 production and cyclooxygenase-2(COX2) activation, ultimately leading to increased production of prostaglandin E2(PGE2). COX2 and PGE2 are potent inducers of aromatase activation (Leyendecker et al., 2009; Leyendecker and Wildt, 2011), and testosterone is aromatized by activated P450, leading to increased E2 synthesis (Zarate-Perez et al., 2018). Progesterone is able to resist the action of estrogen and alter the continuous proliferation of the endometrium. However, the expression of progesterone receptor (PR) is downregulated or absent in the ectopic lesions of AM patients compared with healthy controls, resulting in progesterone resistance. As a result, the proliferative effect of excess estrogen cannot be countered by progesterone (Mehasseb et al., 2011; Thieffry et al., 2022). The increase in estrogen not only induces endometrial proliferation and repair through the estradiol receptor β (ERβ), but also promotes the secretion of oxytocin through the estradiol receptor α (ERα), which continuously stimulates the uterus to be in a state of peristalsis and damage, causing the uterus to enter a vicious cycle of chronic damage, proliferation and inflammation. Excessive peristalsis of the uterus causes destruction of the myometrium and facilitates invasion of the endometrial basalis cells, resulting in the formation of AM (Leyendecker et al., 2009; Leyendecker and Wildt, 2011) (Figure 3A).

Some patients with the Mayer-Rokitansky-Küster-Hauser (MRKH) syndrome have been reported to suffer from AM despite their primordial uteri that lack a functional endometrium (Chun et al., 2013; Pinto et al., 2022). In such AM patients, the formation of ectopic lesions cannot be explained by the theory of invagination. Therefore, the metaplasia theory postulates that AM lesions may also originate from the metaplasia of residual Müllerian ducts (MDs) with differentiation potential within the myometrium. This theory requires the understanding of the concept of “archimetra”, which refers to the endometrium and the subendometrial myometrium. During embryonic development, the archimetra originates from the MDs and undergoes cyclic changes regulated by hormones. On the other hand, the outer muscular layer of the uterus does not develop from the MDs and is called the neometra. Together, the archimetra and neometra make up the uterus (Leyendecker et al., 1998). Residual MDs with differentiation potential can transform into endometrial glands and mesenchyme within the myometrium, resulting in ectopic endometrium (Spencer et al., 2012). This theory could explain the formation of deep infiltrative EM in the rectovaginal septum, where the ectopic lesion originates from the metaplasia of residual MDs (Donnez et al., 1995) (Figure 3B).

Many other factors may contribute to the formation of AM. EMT refers to the process of epithelial to mesenchymal cell transformation, which confers the ability of cell metastasis and invasion. EMT is involved in processes such as embryonic development, organ fibrosis, tissue damage and repair, and tumor metastasis (Nieto et al., 2016; Zhang and Weinberg, 2018). Estrogen can induce EMT through the Notch signaling pathway (Li et al., 2018), Wnt/β-catenin signaling pathway (Zhou et al., 2012), and TGF-β/Smad signaling pathway (Fan et al., 2014), thus promoting AM formation. The decrease in E-cadherin paralleled by the increase in vimentin and N-cadherin is an important feature of EMT and a pattern also observed in AM lesions, indicating that EMT promotes the development of AM (Chen et al., 2010; An et al., 2017) (Figure 3C). Endometrial components have been identified in the myometrial lymphatics, which suggests that lymphatic dissemination may be a pathway of basal endometrial invagination. Guo et al. proposed the concept of endometrial-myometrial interface disruption (EMID) based on the mechanism of wound healing, which is a further refinement and complement to the TIAR mechanism and the above mechanisms (Guo, 2020). Iatrogenic injury and abnormal uterine peristalsis can lead to EMID, platelet aggregation and tissue hypoxia. Consequently, the increase in estrogen promotes excessive uterine peristalsis through ERα and ERβ, and tissue hypoxia activates TGF-β, VEGF, and COX2 signaling pathway (Figure 3A). Furthermore, genetic and immune factors are also involved in the formation of AM. Altogether, these findings demonstrate that AM is a complex and refractory condition that involves the cross-talk and interaction among multiple mechanisms (Figure 3D).

## 5 Relevance of EXOs in AM pathogenesis

Despite its benign nature, cell adhesion, invasion, and angiogenesis are important pathological processes in AM development, with similar biological manifestations as malignant tumors. Intercellular communication is a key feature of tumor progression and metastasis, and EXOs are important mediators of cell migration, proliferation, and angiogenesis in the tumor microenvironment. Mishra et al. (2021) found that endometrial epithelium-derived EVs contain genes that specifically participate in various biological processes that facilitate the pathogenesis of endometrial diseases (e.g., EM, endometritis and endometrial cancer) through regulation of inflammation, angiogenesis, and cell proliferation. Several studies have confirmed that the expression of genes carried by EXOs is also altered in eutopic endometrium compared with normal endometrium (Schjenken et al., 2019; Chen et al., 2020c). Therefore, it is hypothesized that EXOs are involved in the pathogenesis of AM by mechanisms similar to those in tumor development. AM and EM are highly similar in pathogenesis, pathological changes, and symptomatic manifestations, and the two are commonly seen together in clinical practice (Leyendecker et al., 2015; Chapron et al., 2017). There are currently few studies on EXOs in AM. By conducting a comprehensive search and review of English studies of EM, we explored the regulatory role of EXOs and their contents, mainly miRNAs, in pathological alterations in AM.

### 5.1 EXOs promote cell migration and invasion

The pathogenesis of AM is associated with enhanced endometrial cell migration and invasion, and regulation of cell migration and invasion affects the formation of ectopic lesions. EXOs enhance cell invasion by inducing EMT. A recent *in vitro* experiment showed that EXOs derived from ectopic endometrium of AM patients can promote macrophage polarization, and polarized macrophages can induce the EMT process of endometrial epithelial cells (EECs) (Hu et al., 2023). MiRNA-210-3p, a hypoxia-associated miRNA responsive to hypoxia inducible factor-1α (HIF-1α), is highly expressed in EM. EXOs containing miRNA-210-3p induce EMT through the activation of the STAT3 pathway and promote cell metastasis and invasion (Okamoto et al., 2015; Zhang et al., 2019c). In contrast to its oncogenic activity in other cancers, miRNA-10b is minimally expressed in AM lesions. miRNA-10b can directly target ZEB1 and PI3K, induce EMT onset by downregulating E-cadherin expression, and increase Akt phosphorylation to promote invasion by endometrial glandular epithelial cells (Nagathihalli and Merchant, 2012; Guo et al., 2015). Hang et al. (2019) found that downregulation of miRNA-145-5p in ovarian cancer cell-derived EXOs promoted cancer progression. When miRNA-145-5p expression is low, Talin1 overexpression induces EMT through activation of the Wnt/β-catenin pathway, thereby promoting endometrial cell migration and invasion (Wang et al., 2021). Chen et al. isolated EVs from AM patients, and AM-derived extracellular vesicles (AMEVs) induced EMT of EECs and conferred an invasive phenotype to EECs. AMEVs contain 20 EMT-related proteins, of which heat shock protein beta-1 (HSPB1) possesses the highest emPAI value. Downregulation of E-cadherin is correlated with the upregulation of HSPB1, which induces EMT and enhances the invasiveness of EECs (Chen et al., 2020a).

### 5.2 EXOs regulate cell proliferation and apoptosis

Abnormal proliferation and apoptosis of smooth muscle cells (SMCs) in EMI is an important cause of AM. Abnormalities in the EMI structure can alter the diastolic rhythm of the uterus while causing tissue hypoxia and inducing a series of molecular changes (Vannuccini et al., 2017; García-Solares et al., 2018). The HSP family can exert cell proliferation and anti-apoptotic effects by activating the innate immunity and acting as powerful immunomodulators. AM patients have higher HSP60 and HSP70 levels in the eutopic and ectopic endometrium than healthy controls, both of which can be carried by EXOs to promote TLRs and estrogen-mediated inflammation and cell proliferation (Ota et al., 1997; Rérole et al., 2011; Khan et al., 2015; Jiang et al., 2017). Let-7a is a member of the miRNA let-7 family, which is aberrantly expressed and regulates cell proliferation and apoptosis in a variety of diseases. Zhang et al. (2018b) found the downregulation of let-7a in EXOs found in the venous blood of lung cancer patients promoted tumor cell proliferation and thus accelerated cancer progression, a process mediated and regulated by EXOs. Huang et al. (2021) found that let-7a was downregulated in EMI smooth muscle cells, which in turn affected the expression levels of various components of the Hippo-YAP1 axis and promoted smooth muscle cell proliferation. Co-culture of umbilical cord-derived mesenchymal stem cell (UC-MSC)-derived EXOs and endometrial stromal cells (ESCs) enhanced the viability of ESCs and promoted cell proliferation (Lv et al., 2020). MiRNA-21 is known to be involved in cell proliferation, differentiation and apoptosis, and is associated with the proliferation and invasion of many tumor cells (Wang et al., 2020b). MSC-EXOs that carry miRNA-21 can promote cell proliferation and inhibit cell apoptosis by attenuating hypoxia-mediated ER stress and inhibiting p38/MAPK phosphorylation (Chen et al., 2020b). MIR22HG is a lncRNA and an oncogene in many cancers. Both MIR22HG and miRNA-2861 were significantly downregulated in AM patient tissues. Cell proliferation assays revealed that MIR22HG may upregulate miRNA-2861 through demethylation, which in turn downregulates STAT3 and MMP2 and inhibits endometrial cell proliferation, suggesting that MIR22HG and miRNA-2861 overexpression may be potential therapeutic target genes for AM (Yu et al., 2021). Taken together, these data suggest that EXOs can regulate cell proliferation and apoptosis through the proteins and miRNAs they transport, thus participating in the development of AM. Moreover, these findings also hint that certain proteins or miRNAs may be therapeutic targets for AM.

### 5.3 EXOs promote neovascularization

Neovascularization is necessary for endometrial invasion into the myometrium, and COX-2, MMP-2 and VEGF are involved in the process of neovascularization (Tokyol et al., 2009; Huang et al., 2014). COX-2 upregulates VEGF expression mainly through derived prostaglandins (PGs) (Cheng et al., 2004). MMP-2 is involved in the degradation of the ECM, which enables stromal cells to invade blood vessels (Li et al., 2006). VEGF can induce chemokine (C-X-C motif) ligand 1 (CXCL1) expression in endometrial epithelial cells by activating the NF-κB signaling pathway to stimulate neovascularization (Lai et al., 2016). These proangiogenic factors can be transported by EXOs to mediate angiogenesis in AM (Aslan et al., 2019). It was reported that mesenchymal cell-derived EXOs are internalized into endothelial cells, and EXOs isolated from ectopic endometrial mesenchymal cells are enriched in proangiogenic factors, which together suggest that EXOs promote and regulate angiogenesis in a paracrine manner (Harp et al., 2016). When EXOs isolated from endometrial MSCs, which have immunomodulatory and regenerative functions, were co-cultured with mouse embryos, they induced endometrial angiogenesis, vascular differentiation and tissue remodeling by triggering the release of proangiogenic factors such as VEGF and platelet-derived growth factor (PDGF) from the embryos, which in turn facilitated neovascularization in AM lesions (Blázquez et al., 2018). Some researchers also collected endometrial specimens from EM patients, isolated ESCs from them, and then centrifuged to obtain EXOs. It was found that lncRNA HOX transcript antisense RNA (HOTAIR) was upregulated in ectopic endometrial tissue, which could be transported from ESCs to surrounding cells by EXOs. It can not only promote cell proliferation, migration and invasion of ESCs, but also promote angiogenesis after co-culture with human umbilical vein endothelial cells (HUVECs). Overexpression of miRNA-761 can reverse the effect of HOTAIR on ESCs and HUVECs through the HOTAIR/miRNA-761/HDAC1 axis, providing a new therapeutic target for EM (Zhang et al., 2022b). Further studies are warranted to investigate the role of EXOs in these proangiogenic signaling pathways and to examine the effect of blocking these pathways in the treatment of AM.

### 5.4 EXOs promote fibrosis formation

Both AM and EM lesions undergo EMT, fibroblast-to-myofibroblast transdifferentiation (FMT), and smooth muscle metaplasia (SMM) to gradually form fibrosis (Liu et al., 2016a; Shen et al., 2016). The miRNA-29 family is closely linked to the EMT process and was found to inhibit endometrial fibrosis by blocking the TGF-β1/Smad pathway (Li et al., 2016). Wang et al. (2019a) intramuscularly injected EXO-miRNA-29 into mice with renal fibrosis, and found that EXO-miRNA-29 reduced the extent of fibrosis by inhibiting the TGF-β1/Smad pathway. miRNA-214 also plays a role in fibrotic disease. miRNA-214-containing EXOs are produced by ectopic ESCs, and injection of EXOs loaded with miRNA-214 mimics into EM mice reduced the expression of fibrosis-associated proteins (Wu et al., 2018). Cellular communication network factor 2 (CCN2) plays a central role in the development of fibrotic diseases. It was reported that serum EXOs of EM patients have lower miRNA-214-3p and higher CCN2 expression than those of healthy controls, and EXO-miRNA-214-3p can inhibit fibrosis by targeting CCN2 (Zhang et al., 2021). Therefore, it can be speculated that EXOs carrying miRNA-29 and miRNA-214 may inhibit fibrosis in AM by downregulating the expression of fibrosis-related factors. Alternatively, these findings also indicate that the downregulation of the above miRNAs may promote fibrosis in AM.

### 5.5 EXOs regulate immune responses

Several studies confirmed that the pathogenesis of AM is associated with the host immune responses (Kozachenko et al., 2017; Bourdon et al., 2021). Activation of the immune system of AM patients leads to the release of a plethora of cytokines, which induces changes in the local immune microenvironment and ultimately influences the development of AM. The stimulator of interferon genes (STING) pathway is associated with the innate immune response. Compared with eutopic endometrium, STING is upregulated in the epithelial cells of ectopic endometrium, and its expression level is correlated with the extent of intraepithelial lymphocyte infiltration, which are cells that induce a chronic inflammatory response in AM (Qu et al., 2020). In contrast, in a study of mouse skin melanoma cells (B16F10), STING agonist (STINGa) delivered by EXOs recruited more CD8^+^ T cells and induced a more potent antitumor response than STINGa alone, suggesting that EXOs could be used as an effective drug carrier for cancer treatment (McAndrews et al., 2021). The inconsistency in the findings on the STING pathway may be attributed to differences in disease type, cell type and EXOs origin, which make comparisons of the results and conclusions difficult. Immune checkpoints, such as the programmed cell death protein 1 (PD-1) and its ligand 1 (PD-L1), are signaling molecules expressed by immune cells that act as gatekeepers of the immune response (Abril-Rodriguez and Ribas, 2017). Studies have shown that EXOs can carry immune checkpoints, such as T cell immunoglobulin domain and mucin domain-3 (TIM-3) and PD-L1 (Gao et al., 2018; Poggio et al., 2019). Galectin-9 is a ligand for TIM-3, and TIM-3/Galectin-9 expression is upregulated in AM, which in turn suppressed immune responses by negatively regulating T cells (Liu et al., 2016b; Huang et al., 2020). Increased TIM-3 expression in tumor cells delivered by plasma EXOs can negatively regulate antitumor responses and accelerate tumor progression through activation of Galectin-9 (Gao et al., 2018). Overexpression of PD-1/PD-L1 in ectopic and eutopic endometrium may be regulated by high levels of estrogen, leading to EXOs-mediated immune dysfunction (Chen et al., 2018a; Yang et al., 2018; Wu et al., 2019). The above results suggest that EXOs can cause immune disorders in the uterine microenvironment by releasing multiple immune-related proteins, inducing immune escape and promoting the formation of AM lesions. These findings also provide new insights to the use of EXOs in guiding immunotherapy for AM.

## 6 Application prospects of EXOs in AM

The above discussion demonstrates that EXOs are involved in the development of AM, and there are substantial differences in their levels between healthy controls and AM patients, suggesting that EXOs and their contents can be used as diagnostic markers and therapeutic targets for AM.

Pathological tissue biopsy is the gold standard for AM diagnosis, but it cannot be used as a continuous monitoring tool because of its invasive nature. Liquid biopsy refers to the search of biomarkers in body fluids, including circulating tumor cells (CTCs), circulating tumor DNA (ctDNA), free cell RNA, and EXOs. Liquid biopsy is a reliable noninvasive diagnostic tool for many diseases and is now gradually being utilized in clinical practice as a replacement of tissue biopsy (Ignatiadis et al., 2021). It has been reported that EXOs-miRNA is not only involved in the occurrence and development of cancer, but also can be used as a biomarker for cancer diagnosis, prognosis and grading basis. For example, the combination analysis of miRNA-1290 and miRNA-375 in EXOs can predict the overall survival of patients with prostate cancer, reflecting the strong potential of EXOs as biomarkers (Endzelins et al., 2017; Dai et al., 2020). Wu et al. used the weighted correlation network analysis (WGCNA) to validate the effectiveness of EXOs-RNAs as diagnostic biomarkers for EM (Wu et al., 2020). Chen et al. isolated EVs from lesions and peripheral blood of AM patients and found 211 proteins that were co-expressed in two types of EVs. In particular, HSP90A, STIP1 and TAGLN-2 were not expressed in blood EVs from non-AM patients, suggesting that these proteins may serve as potential diagnostic markers for AM (Chen et al., 2022). Based on the above discussion, EXOs and their contents can participate in the development of AM by promoting cell migration, proliferation, neovascularization, and fibrosis. Therefore, EXOs can be used as a noninvasive “liquid biopsy” to assess the health status of the endometrium. In the future, it is expected that EXOs and their contents will be used as an early diagnostic tool for AM, which will facilitate the implementation of reliable and effective prophylactic and therapeutic measures for AM and prevent the development of AM.

**FIGURE 4:**
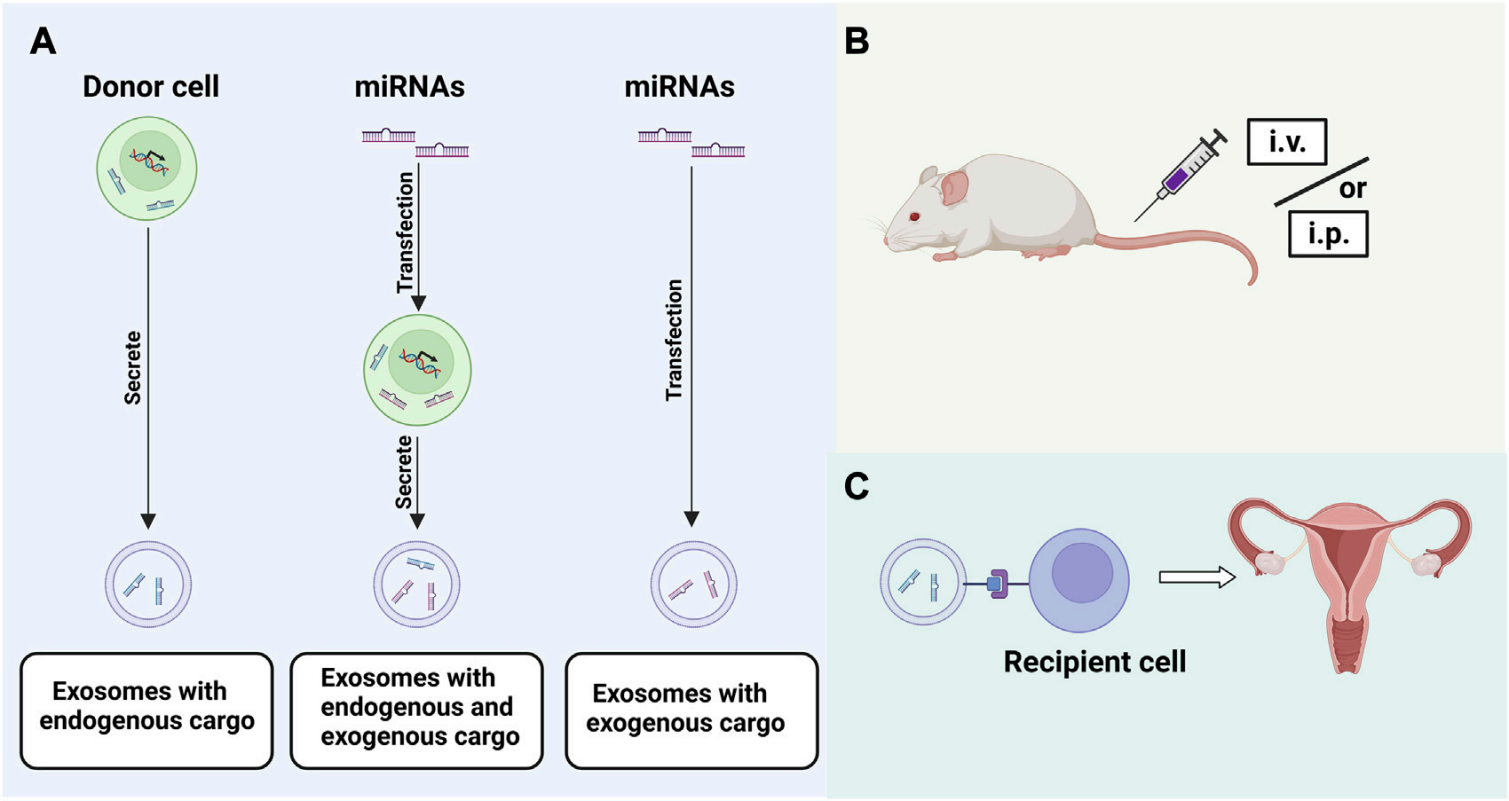
Strategies for EXOs application: **(A)**. Loading of EXOs with exogenous or endogenous miRNAs; **(B)**. Exploration of appropriate route of administration; **(C)**. Targeted binding of EXOs to recipient cells.

EXOs are a natural transport system with many characteristics of an ideal drug carrier, such as long circulating half-life, low immunogenicity and toxicity, inherent ability to target tissues, and good stability and biocompatibility. EXOs can overcome the limitations of most liposomal or polymeric drug delivery systems and can be used therapeutically by overexpressing a kind of content in EXO donor cells or loading an exogenous drug (Turturici et al., 2014; Rani and Ritter, 2016). Other than total hysterectomy, there are no other methods to eradicate AM. MiRNAs, as important cargoes carried by EXOs, can function as biomarkers and therapeutic tools for AM. Xiao et al. found that bone marrow mesenchymal stem cells (BMSCs) in rats suffering from mechanical injury can transfer miRNA-340 to ESCs via EXOs, which can play an antifibrotic role in endometrial diseases and injury (Xiao et al., 2019). CCN2 plays a central role in the development of fibrosis, and EXOs enriched in miRNA-214-3p can downregulate CCN2 expression to inhibit the fibrosis of the endometrium (Zhang et al., 2021). EEC-derived EXOs can inhibit B-cell CLL/lymphoma 9 (BCL9) expression by delivering miRNA-30c to block the Wnt/β-catenin signaling pathway, thereby attenuating the tumor-like behavior of ectopic endometrial epithelial cells (ecto-EECs) in EM (Zhang et al., 2022c). Studies on EXOs in AM treatment are scarce, but EXOs are expected to modulate the relevant pathways for AM treatment by up- or downregulating the expression of EXO contents listed in Table 1. Due to the high targetability of EXOs, their application as a drug carrier has gained great interest from researchers in the treatment of tumors and inflammatory diseases. Curcumin is an anti-inflammatory antioxidant and antitumor agent, but its relative instability and low bioavailability limit its clinical application in the treatment of tumors and inflammatory diseases. Sun et al. (2010) loaded EXOs with curcumin by physical entrapment and found that exosomal curcumin have increased solubility, stability and bioavailability, which promoted the activation of monocytes and hence improved the anti-inflammatory activity of curcumin. Currently, drug loading by EXOs for the treatment of EM-like diseases has not been reported, but related studies in other disease areas have provided insights to the implementation of such studies in the future. Although the therapeutic efficacy of EXOs in AM has not been adequately confirmed in clinical trials, their therapeutic potential should not be underestimated (Figure 4).

**TABLE 1:** The roles of EXOs and their contents in AM.

Contents in EXOs	Pathway	Function	References
miRNA-210-3p	STAT3	Induces EMT and promotes metastatic cell invasion	Okamoto et al. (2015), Zhang et al. (2019c)
miRNA-10b	ZEB1/E-Cadherin PI3K/Akt	Inhibit metastatic invasion of cells	Guo et al. (2015)
miRNA-145-5p	Wnt/β-catenin	Inhibition of EMT and suppression of metastatic cell invasion	Hang et al. (2019), Wang et al. (2021)
HSPB1	HSPB1/E-Cadherin	Induces EMT and enhances endometrial epithelial cell invasion	Chen et al. (2020a)
HSP60, HSP70	TLR4	Promote TLRs, E2-mediated inflammatory response	Ota et al. (1997), Rérole et al. (2011), Khan et al. (2015), Jiang et al. (2017)
let-7a	Hippo-YAP1	Inhibit apoptosis	Zhang et al. (2018b), Huang et al. (2021)
miRNA-21	p38/MAPK	Promote apoptosis	Chen et al. (2020b)
MIR22HG, miRNA-2861	STAT3/MMP2	Inhibits the proliferation of SMCs	Yu et al. (2021)
COX-2	VEGF/COX-2	Promotes p38-mediated cell proliferation	Cheng et al. (2004)
MMP-2	VEGF/MMP-2	Inhibits STAT3- and MMP2-mediated endothelial cell proliferation	Li et al. (2006)
VEGF	NF-κB	Upregulates VEGF-mediated angiogenesis via PGs	Lai et al. (2016)
PDGF	VEGF/PDGF	Participate in degradation of extracellular matrix	Blázquez et al. (2018)
miRNA-761	HOTAIR/miRNA-761/HDAC1	Reversing the effect of HOTAIR on cell proliferation, migration, invasion and angiogenesis	Zhang et al. (2022b)
miRNA-29	TGF-β1/Smad	Accelerates neovascularization	Li et al. (2016)
miRNA-214-3p	miRNA-214/CCN2	Stimulates CXCL1-mediated neoangiogenesis	Zhang et al. (2021)
STING	STING/NF-kB STING/IRF3	Induces endometrial angiogenesis, differentiation and tissue remodeling	Qu et al. (2020)
TIM-3	TIM-3/Galectin-9	Inhibits fibrosis	Liu et al. (2016b), Gao et al. (2018), Huang et al. (2020)
PD-L1	PD-1/PD-L1	Inhibits CCN2-mediated fibrosis process	Chen et al. (2018a), Yang et al. (2018), Wu et al. (2019)

However, in order to realize the diagnosis and treatment function of EXOs, accurate positioning and identification cannot be ignored. The tracking imaging of EXOs *in vivo* helps to determine: 1) The applicability of EXOs as a targeted drug carrier; 2) Role in intercellular communication; 3) The half-life of EXOs. The biological distribution of EXOs can be confirmed by labeling method, which can be divided into exogenous labeling method and endogenous labeling method. Exogenous labeling mainly refers to the surface modification with fluorescent membrane dye such as Dil (Qi et al., 2022), PKH67 (Hu et al., 2022) or PKH26 (Chen et al., 2018b) after extraction. The endogenous labeling is mainly achieved by genetic engineering of EXOs donor cells. The choice of EXOs imaging methods is diverse, such as *in vivo* imaging system, flow cytometry, microscopy, immunohistochemistry, gLuc activity measurement, magnetic resonance, *etc.* How to choose depends on the labeling method and research purpose (Sadovska et al., 2015). The pharmacokinetic study of EXOs is ongoing. However, the efficiency and clearance rate of EXOs transporting content to target cells seem to be related to the nature of the content and the metabolic state of the target cells, which needs to be further clarified. Lai et al. designed a highly sensitive and universal EVs reporting system, which realizes *in vivo* imaging of EVs by biotinylation of EVs with biotin ligase, and can track the biodistribution of EVs and the clearance rate of exogenous EVs (Lai et al., 2014). Fan et al. optimized the EXOs detection biosensor and screened a Raman biosensor. The sensitivity of detecting EXOs through it is adjustable, which helps to achieve differentiated applications. The biosensor can successfully detect tumor with an average diameter of 3.55 mm, which can be used for postoperative tumor recurrence monitoring, and can distinguish tumor patients from healthy subjects, reflecting the clinical application potential of EXOs as diagnostic markers (Fan C. et al., 2021).

## 7 Summary and outlook

EXOs carry a variety of biomolecules, such as lipids, proteins, and nucleic acids. EXOs are released by source cells through exocytosis and then internalized by the target cells through various ways. EXOs can regulate the properties of the target cells, resulting in beneficial and detrimental effects. Therefore, EXOs can be used as a mediator for intercellular bioinformation exchange, and such biological signal changes are involved in the physiological and pathological processes of the organism. In recent years, EXOs have created a great research boom in various disease areas, and their versatility in translational medicine allows them to be used for the diagnosis, prevention and treatment of diseases (He et al., 2018). Based on the aforementioned findings, EXOs can affect the development of AM by regulating cell migration, invasion, proliferation and apoptosis, promoting neovascularization and fibrosis formation, as well as regulating immune responses. EXOs may become a novel tool for the diagnosis and treatment of AM in the future. However, despite research demonstrating the importance of EXOs and the miRNAs, proteins, and other components they contain in the onset, progression, diagnosis, and treatment of AM, their mode of action remains unclear due to the lack of data.

Despite the benefits and potential of this new diagnostic and therapeutic tool, there are still some important concerns that need to be addressed. The biodistribution and pharmacokinetics of EXOs in AM have not been fully understood, and these problems have become a major obstacle to the clinical application of EXOs. Although some miRNAs have been mentioned in this review that can be used for AM treatment through EXOs, the pathogenesis of AM is an extremely complex process that is difficult to regulate simply through a few EXO-miRNAs. Therefore, more highly sensitive EXO-miRNAs that are involved in various aspects of AM or play key roles in different stages of AM will need to be identified. Studies have shown that miRNA-26-5p is significantly downregulated while miRNA-6795-3p is upregulated in EM dysmenorrhea patients. MiRNA-215-5p expression is lower whereas miRNA-6795-3p expression is higher in EM infertile patients than in other EM patients (Wu et al., 2022). The clinical symptoms of AM are equally complex, and there is still a lack of reliable evidence regarding whether the common EXO-miRNAs differ among AM patients with different clinical mresearch directionsanifestations. Studies (Greening et al., 2016) have shown that EXOs of EECs origin are influenced by the hormone levels of the menstrual cycle, and when applying EXO contents for the diagnosis and treatment of AM, the dominant EXOs should be screened according to the menstrual cycle. In the future, it may be possible to select relevant EXOs as drug carriers to exert therapeutic functions in AM and improve the current status of hormonal drug therapy. At present, there are many drug loading methods, which are generally divided into direct loading and transformation of EXO donor cells. The former includes co-incubation of drugs and EXO donor cells, electroporation and EXO transfection, while the latter includes donor cell activation and transfection (Familtseva et al., 2019). However, all forms have their limitations, and new efficient drug-loading methods need to be further developed.

The effectiveness and safety of EXOs as drug carriers still need to be verified. The formation of AM is slow, so blocking or reversing AM also takes a long time, and requires continuous intervention. Therefore, it is clear that a new biocompatible scaffold needs to be designed to extend the biological activity of EXOs for continuous treatment. It should also be noted that there are rhythmic contractions and periodic shedding of the endometrium, so the targeting and stability of the EXOs drug delivery system are particularly important. In any case, the surface engineering of EXOs seems to be an essential part before clinical application. The modification engineering of EXOs is mainly divided into two categories, genetic engineering and chemical modification. Their purpose is to enhance the targeting specificity and therapeutic stability of EXOs delivery systems. Genetic engineering is to fuse the targeting ligand with the EXOs membrane protein, and then the donor cells can produce EXOs showing the targeting ligand. At present, the most widely used membrane proteins are LAMP-2b, lactadherin, and platelet-derived growth factor receptors (PDGFRs) (Barile and Vassalli, 2017). Chemical modification is bioconjugation of targeting ligand with surface proteins (Liang et al., 2021). The surface engineering of EXOs has been carried out in some diseases (Cheng et al., 2018; Zhang et al., 2022a), but has not been reported in the field of AM. In addition, other biological information of the donor cells may be delivered into the target cells/organs in the process of targeted therapy, which may affect the therapeutic effect or even accelerate the disease process. Hence, it is necessary to fully understand the biogenesis and internalization of EXOs and pay attention to the selection of suitable EXO donor cells. And the appropriate drug delivery route also deserves further discussion. At present, intravenous injection and subcutaneous injection are widely used, and intrauterine injection seems to be more suitable for the treatment of AM. Lin et al. treated thin endometrium SD rats by intrauterine injection of EXOs, which could promote endometrial regeneration and improve pregnancy outcome (Lin et al., 2021). This provides a good inspiration for us to explore the appropriate route of administration of AM in the future. Chen et al. (2020a) used differential centrifugation combined with density gradient centrifugation to extract AMEVs. The authors did not separate EXOs from EVs because there was no direct criterion for distinguishing, isolating and identifying subpopulations of cell-derived EVs. Therefore, how to effectively extract AM-derived EXOs and their contents may be a topic of future research.

Although there are many challenges for EXOs to overcome as noninvasive diagnostic tools and drug carriers, nanomedicine is an area that is rapidly evolving. It is hopeful that with continued study into the molecular makeup of EXOs and their contents, as well as the gradual advancement of gynecological molecular biology and genetic engineering techniques, EXOs will become an ideal diagnostic tool for AM and open new avenues to the treatment of AM.
